# Eavesdropping and Jamming Selection Policy for Suspicious UAVs Based on Low Power Consumption over Fading Channels

**DOI:** 10.3390/s19051126

**Published:** 2019-03-05

**Authors:** Xiaoming Wang, Demin Li, Chang Guo, Xiaolu Zhang, Salil S. Kanhere, Kai Li, Eduardo Tovar

**Affiliations:** 1 College of Information Science and Technology, Donghua University, Shanghai 201620, China; xmwang@shea.gov.cn (X.W.); guochang@mail.dhu.edu.cn (C.G.); xiaoludhu@mail.dhu.edu.cn (X.Z.); 2 Engineering Research Center of Digitized Textile and Apparel Technology, Ministry of Education, Shanghai 201620, China; 3 Shanghai Earthquake Administration, Shanghai 200062, China; 4 School of Computer Science and Engineering, UNSW Sydney, Sydney, NSW 2052, Australia; salil.kanhere@unsw.edu.au; 5 CISTER Research Unit, 4200-135 Porto, Portugal; kaili@isep.ipp.pt (K.L.); emt@isep.ipp.pt (E.T.)

**Keywords:** selection policy, eavesdropping, jamming, fading channel, UAV

## Abstract

Traditional wireless security focuses on preventing unmanned aerial vehicle (UAV) communications from suspicious eavesdropping and/or jamming attacks. However, there is a growing need for governments to keep malicious UAV communications under legitimate surveillance. This paper first investigates a new surveillance paradigm for monitoring suspicious UAV communications via jamming suspicious UAVs. Due to the power consumption limitation, the choice of eavesdropping and jamming will reflect the performance of the UAVs communication. Therefore, the paper analyses the UAV’s eavesdropping and jamming models in different cases, and then proposes the model to optimize the data package in the constraints of lower power consumption, which can be solved by the proposed selection policy. The simulation results validate our proposed selection policy in terms of power consumption and eavesdropped packets. In different fading models, power consumption increases with time, regardless of distances, and our proposed policy performs better in Weibull fading channels in terms of eavesdropped packets.

## 1. Introduction

Recently, unmanned aerial vehicle (UAV) techniques have been widely applied to wireless communication systems such emergency rescue, homeland security, etc., owing to the flexible and quick deployment. Researchers from academia, industry, government agencies, etc., have paid lots of attractions to UAV communications. Game theory has been adopted to deal with a smart attacker from UAV [1]. Traditional UAV network security studies generally assume UAV communications are authorized and rightful, so researchers put great efforts to preventing existing UAV communications form malicious attacks such as jamming and eavesdropping [2,3,4,5]. However, the paradigm has changed with the development of UAV technologies. Terrorists or criminals may use UAVs to establish wireless communications for committing crimes and terrorism [6,7]. For instance, the eavesdroppers in the UAV communication networks can overhear the secure message, thus improving the capacity of communication network by reporting faked channel state information on the basis of the continuously changing channel environments [8,9]. More seriously, criminals can use UAV communication networks to commit bombing activities, and business spies may use them to filch trade secrets.

**Figure 1:**
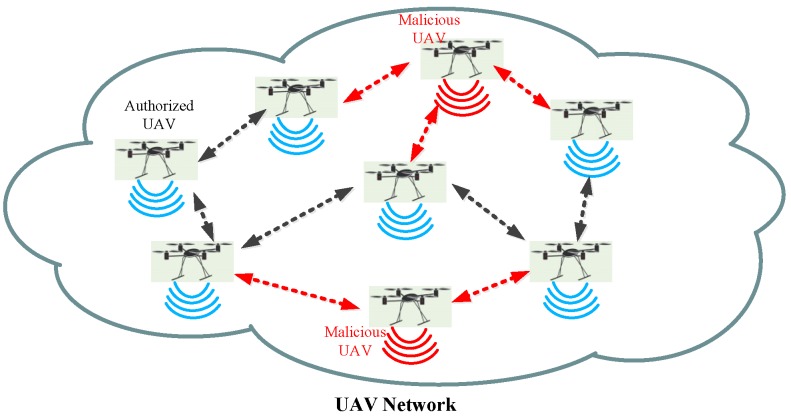
A malicious eavesdropping scenario where malicious unmanned aerial vehicles (UAV) attack authorized UAVs through the UAV network.

In traditional UAV surveillance works, eavesdropping and jamming UAVs are usually static during their tasks, while in this paper, we consider the UAV’s dynamic motion, which can reflect the performance of jamming selection on power consumption. The policy can provide the optimal results of eavesdropping and jamming selection based on power consumption in different locations. As shown in Figure 1, authorized UAVs share information through an existing UAV network, which may change topology occasionally because of UAV’s unpredictable trajectory. The new infrastructure-free mobile communication can be easily used by malicious UAVs (marked as red ones), e.g., criminals, terrorists, and business spies, to commit crimes, jeopardize public safety, invade the secret database of other companies, etc., thus imposing new challenges on the public security [1]. Therefore, there is a growing need for government agencies to legitimately monitor and eavesdrop wireless communications of suspicious UAVs [8].

**Figure 2:**
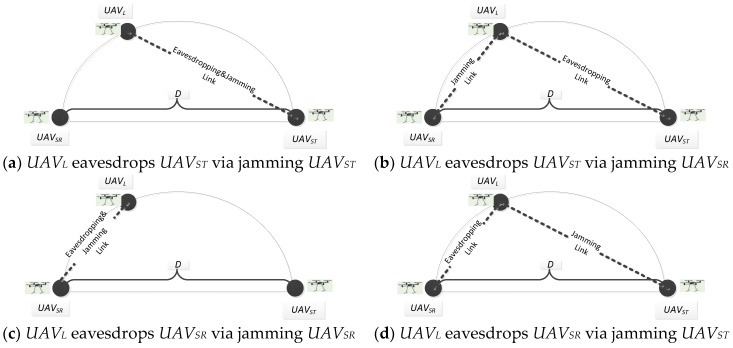
Eavesdropping via jamming.

In particular, we consider four surveillance scenarios as shown in Figure 2, where a legitimate UAV, i.e., *UAV_L_*, aims to monitor a suspicious communication link from a suspicious UAV transmitter (*UAV_ST_*) to a UAV receiver (*UAV_SR_*) over fading channels. It is seldom to have significant multipath links in the sky. However, flying UAVs are strictly restricted according to policies. It is allowed for flying UAVs freely under some low altitudes, which are even lower than tall buildings, and what is more, extreme weather conditions may also influence the state of communication links for UAVs, so there are still scenarios for UAVs communication in multipath links. In reality, UAV transmitter and UAV receiver are relative, since communication links are bi-directional, using a pair of transmitter and receiver for simultaneous transmission in both directions.

In this scenario, we assume that the suspicious pair of UAVs (known as *UAV_ST_* and *UAV_SR_*) has been detected by authorized agencies at the beginning, and they are eavesdropped by a legitimate UAV monitor (*UAV_L_*). Suspicious users’ detection and association can be referred to in Reference [9].

We use the eavesdropping model proposed by Jie Xu, et al. [10] which proactively generate jamming signals to interfere with the suspicious communication link through a full-duplex mode, so as to decrease the achievable data rate at the suspicious transmitter or receiver for overhearing more efficiently.

In order to initialize investigation, we assume that no advanced anti-eavesdropping schemes for security are employed by suspicious UAVs. Based on such assumptions, *UAV_L_* can overhear information successfully from the suspicious UAVs only when the received signal-to-noise ratio (SNR) (and accordingly the achievable data rate) at *UAV_L_* is no smaller than that at *UAV_SR_*, since in this case *UAV_L_* can decode the data that can be decoded at *UAV_SR_* [10]. Let *R_L_* and *R_S_* denote the achievable data rate of the legitimate eavesdropping link form *UAV_ST_* to *UAV_L_* and the communication rate of the suspicious link form *UAV_ST_* to *UAV_SR_*, respectively. Then, *UAV_L_* can decode transmitted signal correctly (with arbitrarily small error) if, and only if, *R_L_* is no smaller than *R_S_*. We define the eavesdropping rate *R_E_* as the suspicious data rate that *UAV_L_* can successfully decode, which is given as *R_E_* = *R_S_* if *R_L_* ≥ *R_S_*, and *R_E_* = 0 if *R_L_* < *R_S_*. *UAV_ST_* and *UAV_SR_* are assumed to fly following a collision-free formation, where they keep a prescribed relative distance and angle. There are four cases for *UAV_L_* to successfully eavesdrop suspicious communication link. Case 1, as shown in Figure 2a, *UAV_L_* eavesdrops suspicious *UAV_ST_* by sending jamming signals to *UAV_SR_*. In this case, *UAV_ST_* increases transmission power in order to sustain *R_S_* at its original level, thus increasing *R_L_* inevitably in the eavesdropping link. When *R_L_* is no smaller than *R_S_*, *UAV_L_* is able to decode the whole information that can be decoded at *UAV_SR_* to fulfill eavesdropping missions. Case 2, as shown in Figure 2b, *UAV_L_* eavesdrops suspicious *UAV_ST_* by sending jamming signals to *UAV_SR_*. Take the time-division-duplex (TDD) multi-antenna transmission scheme as an example, where *UAV_ST_* designs its transmit beamforming vectors based on the reverse-link channel estimation from *UAV_SR_*. In that case, *UAV_ST_* can spoof the reverse-link transmit signals received by *UAV_ST_*, such that *UAV_ST_* estimates a fake channel, and changes its beamforming direction towards *UAV_L_* and away from *UAV_SR_* [11]. This approach increases *R_L_* and decreases *R_S_*, and accordingly improves *R_E_*. Case 3, as shown in Figure 2c, *UAV_L_* eavesdrops suspicious *UAV_SR_* by sending jamming signals to *UAV_SR_*. In that case, *UAV_SR_* increases transmission power in order to sustain *R_S_* at its original level, thus increasing *R_L_* inevitably in the eavesdropping link. When *R_L_* is no smaller than *R_S_*, *UAV_L_* is able to decode the whole information that can be decoded at *UAV_ST_* to fulfill eavesdropping missions. Case 4, as shown in Figure 2d, *UAV_L_* eavesdrops suspicious *UAV_SR_* by sending jamming signals to *UAV_ST_*. Take the time-division-duplex (TDD) multi-antenna transmission scheme as an example, where *UAV_SR_* designs it’s transmit beamforming vectors based on the reverse-link channel estimation from *UAV_ST_*. In that case, *UAV_SR_* can spoof the reverse-link transmit signals received by *UAV_SR_*, such that *UAV_SR_* estimates a suspicious channel, and changes its beamforming direction towards *UAV_L_* and away from *UAV_ST_* [12]. This approach increases *R_L_* and decreases *R_S_*, and accordingly improves *R_E_*.

We have previously discussed the first approach to eavesdrop suspicious communication link by jamming *UAV_SR_*, as shown in Figure 2a [13], so this paper mainly focuses on the other three eavesdropping and jamming cases, as shown in Figure 2b–d. In practice, UAV’s trajectory period depends on the battery charge. Low power consumption can make sure the UAV fly in a relative long period. In this paper, we aim to (1) minimize the power consumption at *UAV_L_*, and to (2) maximize the eavesdropping rate at *UAV_L_*. Specifically, when the constraint of suspicious data rate is given, we formulate an optimization problem to find the most efficient jamming power allocation at *UAV_L_* to maximize the eavesdropping rate, which is polynomially solvable. Moreover, we propose a selection policy to facilitate the simultaneous eavesdropping and jamming for *UAV_L_* on the flight, which also derives the optimal jamming power by using linear programming. In particular, the proposed policy allocates the jamming power over the fading channel according to the limited jamming power constraint, as well as the position of *UAV_L_*. The impacts of fading states on the performance of our policy are analyzed by applying the proposed policy to four common fading models, i.e., Rayleigh, Ricean, Weibull, and Nakagami.

In our paper, we considered the topology between the legitimate UAV and two suspicious UAVs is a semi-circle with a diameter *D*. We mainly consider an optimal policy strategy for the legitimate UAV to obtain a good performance on monitoring. From the analysis, it is clear that the distance between UAVs is the key to the problem. Thus, considering UAVs’ distance is much more meaningful compared to the trajectory design in our model. In fact, the change of trajectories causes the change of distances between legitimate UAV and suspicious UAV, so we can apply our results in various trajectories. The main contributions of this work can be summarized as follows:

(1) Traditional works focused on achieving secure UAV-ground (U2G) communications in the presence of terrestrial eavesdroppers/jammers, while in our paper, we considered UAV-UAV (U2U) communications in the air, so we formulated suspicious UAVs’ distance model, which considered the dynamic mobility of suspicious UAVs in sequence time slots;
(2) Traditional works usually consider one case for eavesdropping and jamming, while in our paper, we proposed four cases of eavesdropping and jamming over fading channels, and then formulated an optimization problem to find the most efficient jamming power allocation at *UAV_L_* to maximize the eavesdropping rate;
(3) Traditional works focus on improving power consumptions or data receive rate respectively, while in our paper, we proposed a selection policy to facilitate the simultaneous eavesdropping and jamming for *UAV_L_* on the flight, which allocated the jamming power over the fading channel according to the limited jamming power constraint as well as the position of *UAV_L_*.

The rest of the paper is organized as follows: Section 2 introduces related works on security techniques in UAV networks. In Section 3, we design the system model on legitimate eavesdropping and jamming. Section 4 proposes the problem formulation and selection policy, as well as the complexity and feasible solution analysis. Simulation results are shown in Section 5, followed by a conclusion in Section 6.

## 2. Related Works

In the literature, there have been a handful of methods for preventing existing wireless networks (e.g., cellular networks) from malicious attacking since wireless networks are prone to malicious attacks such as eavesdropping attack [12], DoS attack [14], spoofing attack [15], MITM attack [16], message falsification/injection attack [17], etc. For instance, authorized devices in a wireless network can, by interference, be illegal devices in the same network in terms of information stealing or virus attacking. Moreover, malicious device may overhear wireless communications sessions, as long as it is within the transmit coverage area of the transmitting device. Generally speaking, the requirements of confidentiality, availability, integrity, and authenticity should be satisfied by secure wireless communications [18]. Cryptographic techniques for preventing eavesdroppers from intercepting data transmissions between legitimate users are typically employed by existing communication systems, thus maintaining confidential transmission in wireless networks [19,20]. For example, passive eavesdropping is applicable to intercept infrastructure-free wireless communications (e.g., UAV networks) [21].

Recently, physical-layer security has emerged as a promising solution to secure UAV communications against eavesdropping attacks [22,23,24,25]. The authors in Reference [22] proposed an algorithm to adaptively control the UAV’s location over time to optimize UAV’s average secrecy rate basing on a secure single-UAV communication system. In Reference [23], authors regarded UAVs as friendly jammers to protect the ground wireless communication, while authors in References [24,25] employed UAVs as mobile relays to facilitate secure or reliable wireless communications. Authors in [26] introduced a power allocation strategy which was regarded as a zero-sum game between the transmitter and the eavesdropper. In Reference [27], authors considered a power control strategy based on Q-learning for the transmitter to enhance the secure capacity via preventing from smart attacks in the dynamic game, however, authors in Reference [27] did not consider the practical channel estimation error, which should not be ignored in the practical communication scenarios, since it will give a significant impact on the network performance. The authors in Reference [28] proposed the optimal power allocation strategies by studying the impact of channel estimation error on the capacity of specific channels. Authors in Reference [29] proposed a theoretical communication scheme, which use multiple antennas to generate artificial noise to degrade the channel quality of eavesdroppers. In Reference [30], authors proposed a low-density parity-check protocol, which used a four-step procedure to ensure wireless information-theoretic security, to achieve communication rates close to the fundamental security limits in wireless communications. However, none of these works [22,23,24,25,26,27,28,29,30] consider the use of proactive eavesdropping to enhance network security.

In order to enhance the quality of secure wireless transmissions, jamming the eavesdropper is an effective approach [31,32,33]. Authors in Reference [31] presented a cooperative jamming scheme, which help a legitimate user improve its data rate via sending a jamming signal to the eavesdropper. The authors in Reference [32] presented a hybrid artificial fast fading scheme, which achieved better performance for eavesdropper. In Reference [33], authors proposed a full-duplex scheme, which transmitted the jamming signal to degrade the channel of eavesdropper. Under this scheme, the system was no longer interference-limited, compared with the half-duplex case. Reference [34] formulated a stochastic game, and provided insights for secret and reliable communication against both jamming and eavesdropping. However, authors in References [31,32,33,34] considered eavesdropping as an illegitimate attack and targeted on decreasing the eavesdropping performance. Authors in References [35,36,37] focused on achieving secure UAV-ground (U2G) communications in the presence of terrestrial eavesdroppers/jammers, they did not consider UAV-UAV (U2U) communications in the air. Reference [12] discussed how an active eavesdropper can attack the training phase in wireless communication to improve its eavesdropping performance, however, Reference [12] did not consider the mobility of UAVs in their communications, and Reference [12] just considered the case of eavesdropping and jamming. In general, there is a lack of researches on power consumption controlling, legitimately eavesdropping and selection policy towards suspicious UAV communications.

## 3. System Model

### 3.1. Assumptions

We consider that the distance between suspicious UAV transmitter (*UAV_ST_*) and receiver (*UAV_SR_*) is denoted as *D* meters. The distance can be calculated in the subsequent time slot, considering the dynamic mobility of the two UAVs. Without loss of generality, we consider legitimate eavesdropper (*UAV_L_*) patrols in a predetermined circular trajectory between *UAV_ST_* and *UAV_SR_* with a diameter *D*, particularly, the wireless link dynamics that are affected by the distance between *UAV_L_* and the suspicious UAVs are identical on a semi-circle of the trajectory. As a result, we consider the trajectory of *UAV_L_* as a semi-circle, even though the distance between *UAV_L_* is dynamic with time-depend.

The suspicious communication between *UAV_ST_* and *UAV_SR_* consists of *m* number of time slots, and each time slot is denoted as *x*. We assume that *UAV_ST_* communicates with *UAV_SR_* in a TDMA fashion, however, it should be noted that our method is generalized and thus agnostic of the MAC protocol in use. In our proposed model, we assume that the suspicious UAVs consider the *UAV_L_*’s eavesdropping signal as interference during the wireless communication.

In fact, our policy proposed in Section IV is general and can support other shapes of flight trajectory since we have considered different fading channels with path loss that is affected by the distance between hostile UAV pairs, regardless of trajectories of UAVs. Moreover, Table 1 lists the fundamental variables that have been used in our system model.

### 3.2. Suspicious UAVs’ Distance Model

The distance between *UAV_L_* and *UAV_ST_*, and the distance between *UAV_L_* and *UAV_SR_* relate to the performance of eavesdropping and jamming. Therefore, we will discuss the suspicious UAVs’ distance model in this part, which is based on the position of *UAV_L_* and the suspicious UAVs’ dynamic mobility.

**Figure 3:**
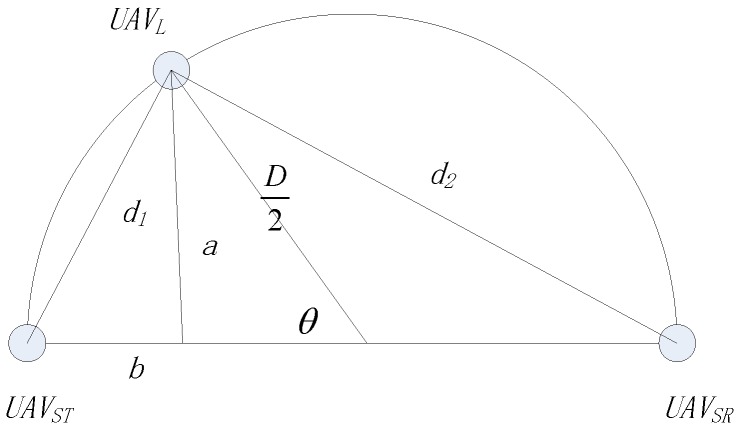
The illustration of distance when θ<π/2.

As shown in Figure 3, the distance between *UAV_L_* and *UAV_ST_* at time slot x, which was denoted as $d_{1}\left(x\right)$, can be described as:

(1) $$d_{1}\left(x\right)=\sqrt{{\left(\frac{D}{2}-\frac{D}{2}\cos\theta \left(x\right)\right)}^{2}+{\left(\frac{D}{2}\sin\theta \left(x\right)\right)}^{2}}=\frac{\sqrt{2}D}{2}\sqrt{1-\cos\theta \left(x\right)}$$

Additionally, the distance between *UAV_L_* and *UAV_SR_*, $d_{2}\left(x\right)$, is given by $d_{2}\left(x\right)=\sqrt{D^{2}-d_{1}^{2}\left(x\right)}$. Note that $d_{1}\left(x\right)$ and $d_{2}\left(x\right)$ can be also estimated by other ways, e.g., measuring receiving signal strength, or signal angle of arrival of *UAV_ST_* or *UAV_SR_*.

**Figure 4:**
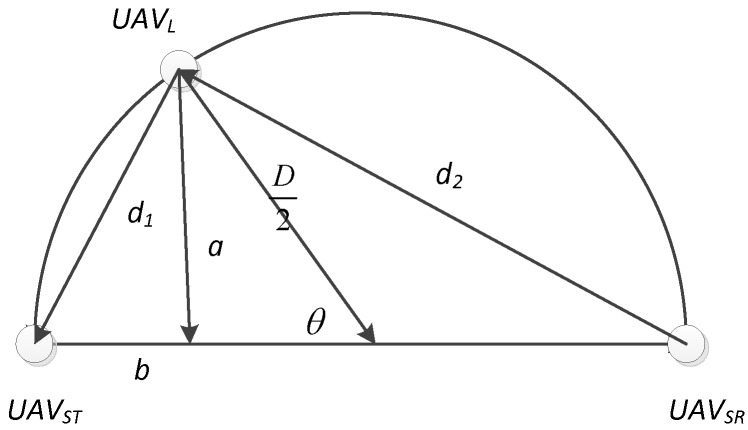
The illustration of distance when θ>π/2.

The angle variation θ(x) depends on the real-time position of *UAV_L_*. However, as shown in Figure 4, the results of $d_{1}\left(x\right)$ is the same as Equation (1), because the expression of variations a and b can be transformed under the condition of θ<π/2, which means that

(2) $$a=\frac{D}{2}\sin\left(\pi -\theta \left(x\right)\right)=\frac{D}{2}\sin\theta \left(x\right),b=\frac{D}{2}+\frac{D}{2}\cos\left(\pi -\theta \left(x\right)\right)=\frac{D}{2}-\frac{D}{2}cos\theta \left(x\right)$$

**Figure 5:**
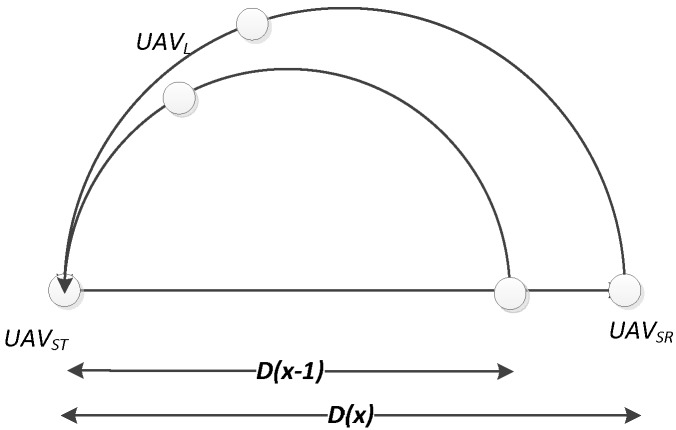
The illustration of dynamic mobility of suspicious UAVs.

The model is two-dimensional, and considers the dynamic mobility of suspicious UAVs in sequence time slots, as shown in Figure 5. The distance variation D is improved as a dynamic variation that relates to the time slot,

(3) D(x)=D(x−1)+φ∆v

Here, φ is the duration of each time slot, and ∆v is a vertex that presents the speeds’ difference value of *UAV_ST_* and *UAV_SR_*. We do not include three-dimensional degrees of freedom for improving the security, but that will be our future works.

### 3.3. Eavesdropping and Jamming Model

Based on the power constraint of UAVs, the suspicious UAVs’ selection for eavesdropping and jamming is an important parameter to be considered in the following algorithm. The optimal selection depends on the *UAV_L_*’s position at time slot x. There are four cases as follows:

**Case 1:***UAV_L_* eavesdrops and jams *UAV_ST_*.

As shown in Figure 2a, *UAV_L_* only chooses *UAV_ST_* for eavesdropping and jamming. According to References [19,38], at time slot *xth*, the channel gain from *UAV_ST_* to *UAV_SR_*, which was denoted as $H_{s}\left(x\right)$, is expressed as:

(4) $$H_{s}\left(x\right)=\frac{\lambda H_{s}\left(x-1\right)+n\sqrt{1-\lambda^{2}}}{D^{\alpha_{2}}}$$

where $\alpha_{2}$ denotes the path-loss exponent in the suspicious link and λ presents the coefficient which adjusts two components: the weights of the auto-correlated and the independent. *n* is a Gaussian random number generated by Additive White Gaussian Noise (AWGN). For the suspicious communication link, we define Signal to Interference plus Noise Ratio (SINR) at *UAV_ST_* at time slot *x* as $\gamma_{s}\left(x\right)$, which is given by

(5) $$\gamma_{s}\left(x\right)=\sqrt{\frac{H_{s}\left(x\right)\cdot K_{2}^{-1}\ln\frac{K_{1}}{\epsilon }\cdot \left(2^{\rho \left(x\right)}-1\right)}{N_{0}+P_{L}\left(x\right)}}$$

where ρ(x) denotes the adaptive modulation and coding (AMC) rate of the *UAV_ST_* at time slot *x*, and the highest mode is denoted by $\rho_{M}$. $K_{1}$ and $K_{2}$ are two constants related to the channel. $N_{0}$ denotes the power of white Gaussian noise. ϵ is the required instantaneous bit error rate. As elaborated in the assumption part, the suspicious UAVs consider the *UAV_L_*’s eavesdropping signal as interference during the wireless communication. Hence, the eavesdropping power at time slot x is a part of interference in suspicious communication. Another part of interference is the jamming power from *UAV_L_*. Therefore, the interference power at time slot *x* is denoted as $P_{E}\left(x\right)+P_{J}\left(x\right).$ Likewise, at time slot *x*, the channel gain in the eavesdropping and jamming links, i.e., from *UAV_ST_* to *UAV_L_*, is given by

(6) $$H_{e}\left(x\right)=H_{j}\left(x\right)=\frac{\lambda H_{e}\left(x-1\right)+n\sqrt{1-\lambda^{2}}}{d_{1}^{\alpha_{1}}\left(x\right)}$$

where *n* is a Gaussian random number generated by AWGN. $\alpha_{1}$ denotes the path-loss exponent. $d_{1}\left(x\right)$ is the distance between *UAV_L_* and *UAV_ST_* at time slot *x*, which can be acquired by Equation (1).

As the relative position of *UAV_L_* to *UAV_ST_*/*UAV_SR_* changes from time to time, there are two components in the eavesdropping link, which named as auto-correlated component and independent component. The former relies on the previous channel condition and the latter is independent of previous channels. The two components are adjusted by a coefficient λ. Moreover, λ decreases with the growth of the speed of *UAV_L_*. We define Signal to Noise Ratio (SNR) of the eavesdropping and jamming links at time slot *x* as $\gamma_{e}\left(x\right)$, which is

(7) $$\gamma_{e}\left(x\right)=\gamma_{j}\left(x\right)=\sqrt{\frac{H_{e}\left(x\right)\cdot K_{2}^{-1}\ln\frac{K_{1}}{\epsilon }\cdot \left(2^{\rho \left(x\right)}-1\right)}{N_{0}}}$$

According to the regression model proposed in Reference [20], the PRR of suspicious data packets eavesdropped by *UAV_L_*, which was denoted as R(x), is given by

(8) $$R\left(x\right)={\left(1-\frac{1}{2}exp^{-\beta_{0}\gamma_{e}\left(x\right)+\beta_{1}}\right)}^{8\left(2f-l\right)}$$

where $\beta_{0}$ and $\beta_{1}$ are two constants in the regression model. Moreover, $\beta_{0}$ controls the shape of the regression curve and $\beta_{1}$ induces horizontal shifts of the curve. *f* and *l* denote frame size and preamble size of the data packet, respectively.

**Case 2:***UAV_L_* eavesdrops *UAV_ST_* by jamming *UAV_SR_*.

As shown in Figure 2b, *UAV_L_* chooses *UAV_ST_* for eavesdropping and *UAV_SR_* for jamming. In this case, the channel gain in the eavesdropping link is the same as in Equation (6), and because of the jamming object selection of *UAV_SR_*, the channel gain in the jamming link is changed as:

(9) $$H_{j}\left(x\right)=\frac{\lambda H_{j}\left(x-1\right)+n\sqrt{1-\lambda^{2}}}{d_{2}^{\alpha_{1}}\left(x\right)}$$

where $d_{2}\left(x\right)=\sqrt{D{\left(x\right)}^{2}-d_{1}^{2}\left(x\right)}$. Accordingly, the Signal to Noise Ratio (SNR) in the jamming link denotes as:

(10) $$\gamma_{j}\left(x\right)=\sqrt{\frac{H_{j}\left(x\right)\cdot K_{2}^{-1}\ln\frac{K_{1}}{\epsilon }\cdot \left(2^{\rho \left(x\right)}-1\right)}{N_{0}}}$$

**Case 3:***UAV_L_* eavesdrops and jams *UAV_SR_*.

As shown in Figure 2c, *UAV_L_* only chooses *UAV_ST_* for eavesdropping and jamming. The channel gains for eavesdropping and jamming links are denoted as:

(11) $$H_{e}\left(x\right)=H_{j}\left(x\right)=\frac{\lambda H_{e}\left(x-1\right)+n\sqrt{1-\lambda^{2}}}{d_{2}^{\alpha_{1}}\left(x\right)}$$

where $d_{2}\left(x\right)=\sqrt{D{\left(x\right)}^{2}-d_{1}^{2}\left(x\right)}$. Accordingly, the Signal to Noise Ratio (SNR) in the jamming link is the same as in Equation (7).

**Case 4:***UAV_L_* eavesdrops *UAV_SR_* by jamming *UAV_ST_*.

As shown in Figure 2d, *UAV_L_* chooses *UAV_ST_* for jamming and *UAV_SR_* for eavesdropping. In this case, the channel gain in the eavesdropping link is the same as in Equation (11), and the channel gain in the jamming link is the same as in Equation (6).

## 4. Formulation and Policy

### 4.1. Problem Formulation

Without loss of generality, we consider the wireless communication, as shown in Figure 2b for the problem formulation, where *UAV_L_* aims to eavesdrop data packets from *UAV_ST_* via jamming *UAV_SR_*. Note that our algorithm is common in the other three cases because channel gains for eavesdropping links are associated with D(x) according to Equation (11). D(x) is the only parameter that influences eavesdropped data packets. Based on the notations in the system model, we formulate the optimization problem to maximize the eavesdropped data packets via optimizing jamming power. Assume that each suspicious data packet has *b* bytes and then successfully eavesdropped data (in bytes) can be calculated as $\sum_{x=1}^{m}b\cdot R\left(x\right)$ in *m* time slots. To prevent legitimate jamming and eavesdropping being detected by suspicious UAVs, SINR of the suspicious link has to be maintained at a certain threshold δ, which presents $\gamma_{s}\left(x\right)=\delta$. Specifically, the modulation of *UAV_ST_* that is used to transmit data to *UAV_SR_* is $2^{\rho \left(x\right)}$ Quadrature Amplitude Modulation (QAM), where $\rho \left(x\right)=\left\{1,\ldots ,\rho_{max}\right\}$. $\rho_{max}$ indicates the number of modulation levels available for rate adaptation. Constraint $0\leq \sum_{x=1}^{m}P_{L}\left(x\right)\leq P_{L}^{total}$ specifies that the total consuming power (eavesdropping plus jamming) of *UAV_L_* during the eavesdropping period is required to be less than the total obtained power of the *UAV_L_*, $P_{L}^{total}$. Constraint $P_{L}\left(x\right)\leq P_{L}^{max}\;\left(\forall x,\;x=1,2,\ldots ,m\right)$ specifies that, in each eavesdropping period, *UAV_L_* consumes no more than $P_{L}^{max}$ power. Then, the formulation of the problem is presented as follows.

(12) $$\underset{P_{L}\left(x\right),\rho \left(x\right)}{\max}\sum_{x=1}^{m}b\cdot R\left(x\right)$$

Subject to:

(13) $$\gamma_{s}\left(x\right)=\delta$$

(14) $$0\leq \sum_{x=1}^{m}P_{L}\left(x\right)\leq P_{L}^{total}$$

(15) $$P_{L}\left(x\right)\leq P_{L}^{max}\;\left(\forall x,\;x=1,2,\ldots ,m\right)$$

(16) $$1\leq \rho \left(x\right)\leq \rho_{max}$$

Furthermore, in terms of Equation (13), we have

(17) $$\rho \left(x\right)=\log_{2}\left(\frac{\delta^{2}\left(N_{0}+P_{L}\left(x\right)\right)}{H_{s}\left(x\right)\cdot K_{2}^{-1}\ln\frac{K_{1}}{\epsilon }}+1\right)$$

which indicates that the modulation level is adapted by *UAV_ST_* in terms of the consuming power $P_{L}\left(x\right)$ of *UAV_L_*. Specifically, *UAV_ST_* increases ρ(x) to transmit data with an increasing $P_{L}\left(x\right)$ so that SINR of the suspicious link at time slot *x* is maintained at δ. Moreover, considering Equation (5) and Equation (13), the upper bound and the lower bound of the consuming power $P_{L}\left(x\right)$ can be obtained by

(18) $$P_{L}\left(x\right)=\{\begin{array}{c} \frac{H_{s}\left(x\right)\cdot K_{2}^{-1}\ln\frac{K_{1}}{\epsilon }}{\delta^{2}}-N_{0}\;if\;\rho \left(x\right)=1\\ \frac{\left(2^{\rho_{max}}-1\right)H_{s}\left(x\right)\cdot K_{2}^{-1}\ln\frac{K_{1}}{\epsilon }}{\delta^{2}}-N_{0}\;if\;\rho \left(x\right)=\rho_{max} \end{array}$$

Consequently, by substituting Equations (6), (7), (8), (9), (10), and (11) into (13), (14), (15), and (16) the optimization problem is reformulated as follows:

**Optimal Eavesdropping and Jamming Problem:**

$$\underset{P_{L}\left(x\right)}{\max}b\cdot \overset{m}{\underset{x=1}{\sum }}{\left(1-\frac{1}{2}exp^{\beta_{1}-\beta_{0}\delta \sqrt{\frac{H_{e}\left(x\right)+H_{j}\left(x\right)}{H_{s}\left(x\right)}\cdot \left(1+\frac{P_{L}\left(x\right)}{N_{0}}\right)}}\right)}^{8\left(2f-l\right)}$$

Subject to:

$$\begin{array}{c} 0\leq \sum_{x=1}^{m}P_{L}\left(x\right)\leq P_{L}^{total}\\ P_{L}\left(x\right)\leq P_{L}^{max}\;\left(\forall x,\;x=1,2,\ldots ,m\right)\\ P_{L}\left(x\right)\geq \frac{H_{s}\left(x\right)\cdot K_{2}^{-1}\ln\frac{K_{1}}{\epsilon }}{\delta^{2}}-N_{0}\\ P_{L}\left(x\right)\leq \frac{\left(2^{\rho_{max}}-1\right)H_{s}\left(x\right)\cdot K_{2}^{-1}\ln\frac{K_{1}}{\epsilon }}{\delta^{2}}-N_{0} \end{array}$$

### 4.2. Selection Policy For Eavesdropping and Jamming

First, the optimal consuming power, $P_{L}^{*}\left(x\right)$ in the optimization problem is able to be derived by linear optimization techniques, e.g., linear programming. Next, we propose the selection policy to allocate jamming power for *UAV_L_* in real time, as shown in Policy 1. According to Reference [10], *UAV_L_* overhears the channels of suspicious and eavesdropping link via channel probing, so the channel gains $H_{s}\left(x\right)$, $H_{e}\left(x\right)$, $H_{j}\left(x\right)$ and $N_{0}$ are known by *UAV_L_* at the beginning of time slot *x*. Since $\gamma_{s}\left(x\right)=\delta$ is required by *UAV_L_* to successfully eavesdrop the suspicious transmission, we have

$$P_{L}\left(x\right)\geq \frac{N_{0}\cdot \left(H_{s}\left(x\right)-H_{e}\left(x\right)-H_{j}\left(x\right)\right)}{H_{e}\left(x\right)+H_{j}\left(x\right)}$$

where ρ(x) is given by Equation (11). Therefore, the jamming power at *x* = *k* is initialized as

$$P_{L}^{0}\left(k\right)=\frac{N_{0}\cdot \left(H_{s}\left(x\right)-H_{e}\left(x\right)-H_{j}\left(x\right)\right)}{H_{e}\left(x\right)+H_{j}\left(x\right)}$$

Next, initialized jamming and eavesdropping power $P_{L}^{0}\left(k\right)$ is examined by *UAV_L_* if the four constraints in the optimization problem are satisfied. Specifically, if one of the constraints does not hold, it indicates that the required jamming power is much higher than the optimal solution, i.e., the link quality of the eavesdropping link is too low to decode the suspicious packet. In this case, *UAV_L_* does not send the jamming signal to suspicious UAVs for the purpose of power efficiency. Moreover, if $\sum_{x=1}^{k-1}P_{L}\left(x\right)+P_{L}^{0}\left(k\right)\leq P_{L}^{max}$ and constraints (14), (15), and (16) hold, the optimization problem is derived by *UAV_L_*, and the optimal consuming power $P_{L}^{*}\left(x\right)$ is obtained.

**Policy 1** Selection Policy1:
**BEGIN:**
2:k: denotes the current time slot, x: denotes the duration of time slot.3:INPUT: $D\left(0\right),\;n,\;\lambda ,\alpha ,\;\alpha_{2},\Delta v$4:
**If**
∆v=0
**then**
5:

D=D(0)
6:
**Else**
7:
D(k)=D(k−1)=kx∆v
8:
**End if**
9:Acquire: $H_{s}\left(k\right),\;\gamma_{s}\left(k\right)$ via D(k)10:Acquire: *UAV_L_*’s position: $d_{1}\left(k\right),\;d_{2}\left(k\right)$11:**While**    $E\left(k\right)={\left[0,1\right]}^{T}\left||E\left(k\right)={\left[1,0\right]}^{T}|\right|J\left(k\right)={\left[0,1\right]}^{T}||J\left(k\right)={\left[1,0\right]}^{T}$ **do**12:
 Acquire: $P_{L}\left(k\right)=P_{L}^{e}\left(k\right)+P_{L}^{j}\left(k\right)$13:
 power set in all cases: $\left\{P_{L}^{i}\left(k\right)\right\},\;i=1,2,3,4.$14:
**End while**
15:**For**i=1:4, i++ do16:
 **If** the Equations (13) (14) (15) **then**17:
  derive Power-efficient package rate maximum problem18:
  Acquire $P_{L}^{i*}\left(k\right)$19:
 **else**20:

$P_{L}^{i*}\left(k\right)=0,\;E\left(k\right)={\left[0,0\right]}^{T},\;J\left(k\right)={\left[0,0\right]}^{T}$
21:
 **Endif**22:
**endfor**
23:
$P_{L}^{*}\left(k\right)=\min\left\{P_{L}^{i*}\left(k\right)\right\},\;i^{*}=\arg\min\left\{P_{L}^{i*}\left(k\right)\right\}$
24:Output: $E\left(k\right)=E^{i*}\left(k\right),\;J\left(k\right)=J^{i*}\left(k\right)$25:**If**E(k)=E(k−1)&&J(k)=J(k−1) then26:
 *UAV_L_* doesn’t shift the eavesdropping-jamming model.27:
**else**
28:
 *UAV_L_* shifts the eavesdropping-jamming model from E(k−1), J(k−1) to E(k), J(k)29:
**endif**
30:
k=k+1
31:Go back to line 6 until k=m+132:
**END**


### 4.3. Policy Analysis

#### 4.3.1. Computing Complexity

Note that the power consumption of executing selection policy is much smaller than the jamming power of *UAV_L_*, which is negligible. The time complexity of selection policy is denoted as $O\left(n^{2}m+nm\right)$. Based on [13], the time complexity of Power Efficient Legitimate Eavesdropping (PELE) that calculate the optimal power result is O(m) which depends on the number of time slots. Considering the number of cases used in eavesdropping and jamming models, which are denoted as n, the selection policy’s time consumption in finding optimal power solutions is O(nm). After calculating optimal power consumptions in all cases in each time slot, the algorithm uses the Bubble method [39] to acquire the minimum power in all cases, which are denoted as $O\left(n^{2}\right)$ in each time slot and $O\left(mn^{2}\right)$ in the whole eavesdropping and jamming process.

Therefore, the selection policy’s time complexity can be denoted as $O\left(n^{2}m+nm\right)$, where n denotes the number of cases and m denotes the number of time slots.

In our research, we find that it is a challenging problem to solve the optimal number of time slots for accurate resolution of the optimization problem. As the complexity increases, it is really difficult to obtain the optimal number of slots for accurate resolution of our problem. Due to the limitations on laboratory equipment, we only discuss the algorithm performance with six time slots in our simulations. Our further research is to design an algorithm to research the optimal number of slots for accurate resolution of the optimization problem.

#### 4.3.2. Feasible Solution

Regarding the proposed Optimal Eavesdropping and Jamming Problem, we will discuss whether it has the feasible solution or not. Based on Reference [40], the optimization model that has the feasible solution should satisfy three constraints: (a) The variable is effective collection based on the constraints in the optimization model, (b) the objective of the optimization model is the continuous function, and (c) the objective of the optimization model is a convex function. We will prove these three properties in this part.

First, we will discuss the variable’s effective collection under the constraints in our proposed optimization model. The constraints $0\leq \sum_{x=1}^{m}P_{L}\left(x\right)\leq P_{L}^{total}$ and $P_{L}\left(x\right)\leq P_{L}^{max}\;\left(\forall x,\;x=1,2,\ldots ,m\right)$ relates to the practice in the reality, which defines $P_{L}\left(x\right)$’s maximums of upper and lower bound. The last two constraints should be proved, satisfying the effective collection. They make further definition of $P_{L}\left(x\right)$’s upper and lower bound, furthermore, the relationship between $\frac{H_{s}\left(x\right)\cdot K_{2}^{-1}\ln\frac{K_{1}}{\epsilon }}{\delta^{2}}-N_{0}$ and $\frac{\left(2^{\rho_{max}}-1\right)H_{s}\left(x\right)\cdot K_{2}^{-1}\ln\frac{K_{1}}{\epsilon }}{\delta^{2}}-N_{0}$ should be considered. In fact, the parameters $H_{s}\left(x\right)$, $K_{2}^{-1}$, and $\delta^{2}$ are larger than zero. $K_{1}$ is larger than ϵ, which means that $\ln\frac{K_{1}}{\epsilon }>0$, then the last two constraints can be transformed into:

$$1\leq \frac{\delta^{2}P_{L}\left(x\right)}{H_{s}\left(x\right)\;K_{2}^{-1}\ln\frac{K_{1}}{\epsilon }}\leq 2^{\rho_{max}}-1$$

$\rho_{max}$ is a parameter that is larger than 1. Therefore, the variable $P_{L}\left(x\right)$ has the effective collection under the four constraints in the optimization model.

Second, we will discuss the objective’s consecutiveness in the optimization model. Obviously, the objective is a composite function, which uses the constant function, power function, exponential function and the logarithmic function based on $P_{L}\left(x\right)$, $H_{s}\left(x\right)$, $H_{e}\left(x\right)$, and $H_{j}\left(x\right)$. It is easy to prove that the functions of $P_{L}\left(x\right)$, $H_{s}\left(x\right)$, $H_{e}\left(x\right)$, and $H_{j}\left(x\right)$ are all continuous functions. Moreover, the sum function does not affect the function’s consecutiveness. Therefore, the objective in our proposed Optimal Eavesdropping and Jamming Problem is a continuous function.

Finally, we will discuss whether the objective in our proposed Optimal Eavesdropping and Jamming Problem is a convex function or not. In order to simplify, we define the objective function as G(x), where

$$G\left(x\right)={\left(1-\frac{1}{2}exp^{\beta_{1}-\beta_{0}\delta \sqrt{\frac{H_{e}\left(x\right)+H_{j}\left(x\right)}{H_{s}\left(x\right)}\cdot \left(1+\frac{P_{L}\left(x\right)}{N_{0}}\right)}}\right)}^{8\left(2f-l\right)}$$

We have proved that the objective is a continuous function in the above paragraph, and then the convex property can be proved by the second derivation, which is denoted as:

$$G^{\prime \prime }\left(x\right)=-bln\left[8\left(2f-l\right)\right]\cdot \frac{1}{2}\exp\left(\beta_{1}-\beta_{0}\delta \sqrt{\frac{H_{e}\left(x\right)+H_{j}\left(x\right)}{H_{s}\left(x\right)}\cdot \left(1+\frac{P_{L}\left(x\right)}{N_{0}}\right)}\right)\;\cdot ln\frac{1}{2}\left[{\left(\frac{H_{e}\left(x\right)+H_{j}\left(x\right)}{H_{s}\left(x\right)}\right)}^{\prime \prime }+\left(\frac{P_{L}\left(x\right)}{N_{0}}\frac{H_{e}\left(x\right)+H_{j}\left(x\right)}{H_{s}\left(x\right)}\right)\prime \prime \right]$$

According to the non-negativity of exponential function, the second term of $G^{\prime \prime }\left(x\right)$ will be larger than zero. Regarding the first term of $G^{\prime \prime }\left(x\right)$, the preamble size l is always smaller than the frame size f in the practice, then the result of 8(2f−l) will be larger than 1, thus the first term is smaller than 0. Regarding the third term of $G^{\prime \prime }\left(x\right)$, which is denoted as:

$$\begin{array}{cc} {\left(\frac{H_{e}\left(x\right)+H_{j}\left(x\right)}{H_{s}\left(x\right)}\right)}^{\prime \prime } & \\ & \frac{1}{N_{0}}[2P_{L}^{\prime }\left(x\right){\left(\frac{H_{e}\left(x\right)+H_{j}\left(x\right)}{H_{s}\left(x\right)}\right)}^{\prime }+P_{L}\left(x\right){\left(\frac{H_{e}\left(x\right)+H_{j}\left(x\right)}{H_{s}\left(x\right)}\right)}^{\prime \prime }\\ & +P_{L}^{\prime \prime }\left(x\right)\left(\frac{H_{e}\left(x\right)+H_{j}\left(x\right)}{H_{s}\left(x\right)}\right)]\geq 0 \end{array}$$

Therefore, the first term $G^{\prime \prime }\left(x\right)$ is smaller than zero, and the second and the third terms are larger than zero. The second derivate result is smaller than zero. The objective of our proposed Optimal Eavesdropping and Jamming Problem is a convex function.

Finally, from the discussions above, we have the conclusions that: (1) The time complexity of selection policy is $O\left(n^{2}m+nm\right)$, and (2) our proposed Optimal Eavesdropping and Jamming Problem has the feasible solution.

## 5. Numerical Results

In this section, we provide simulation results to verify the performance of our proposed selection policy. Furthermore, we choose four normal fading channels, e.g., Rayleigh, Ricean, Weibull, and Nakagami, to investigate the impacts on our proposed selection policy.

### 5.1. Simulation Configurations

The distance between the two suspicious UAVs is *D*, which various from 500 m to 2000 m, and the path length of *UAV_L_* is πD/2. The patrolling speed of *UAV_L_* is set to 10 m/s. In fact, we do realize the policies for using UAVs in our country. It is allowed for flying UAVs freely under altitudes of 120 m. In our research, the distance variation (from 500 m to 2000 m) is mainly in the same altitude, which can be within the permission of policies. We use MATLAB to conduct the experiments instead of an actual simulator, however, the experiments can be legally carried if there are enough equipped UAVs. The detailed system-level simulation parameters are shown in Table 2.

*UAV_ST_* communicates with *UAV_SR_* in a TDMA fashion for suspicious collision-free transmission. Especially, we consider that a TDMA frame contains 6 time slots, and each of which is 10 s long. In one time slot, *UAV_ST_* transmits its data to *UAV_SR_*, where *UAV_L_* eavesdrops and decides to jam the suspicious communication according to the selection policy. In addition, the suspicious link, eavesdropping link, and jamming link are assumed to be block-fading, i.e., the channels remain unchanged during each transmission block, and may change from block to block.

### 5.2. Eavesdropping Rate and Power Consumption

For comparison, we consider other two legitimate eavesdropping strategies: proactive eavesdropping with constant jamming power and zero jamming power. For the former scheme, we set the constant jamming power to 10^−8^ W (in fact, the constant jamming power can be set to any value below $P_{L}^{max}$, which has little effects on simulation results as observed in the performance). For the latter scheme, we set the constant jamming power to 0, which means *UAV_L_* passively overhears the packets transmitted by suspicious UAVs without sending jamming signal to the suspicious link [17,18,21].

**Figure 6:**
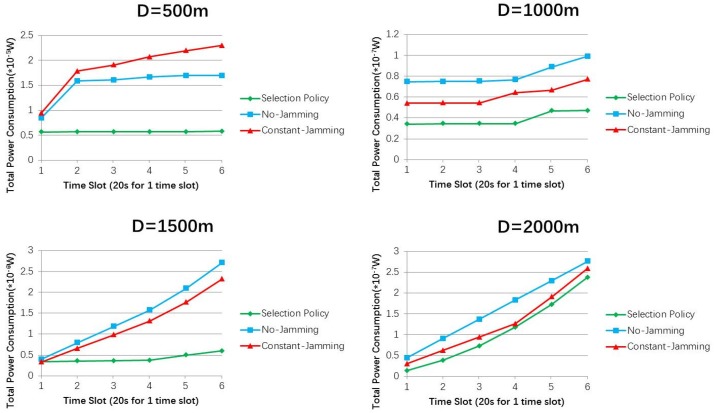
Total power consumptions by *UAV_L_* in different Ds with different jamming methods.

Figure 6 shows that selection policy saves 65.79%, 52.66%, 78.12%, and 13.92% more power than the constant-Jamming scheme, when D = 500 m, 1000 m, 1500 m, and 2000 m, respectively. Selection policy saves 74.73%, 39.02%, 74.35%, and 8.40% more power than the No-Jamming scheme, when D = 500 m, 1000 m, 1500 m, and 2000 m, respectively. The power consumption of selection policy increases as time goes on in each simulation. The reason is that *UAV_L_* consumes power to eavesdrop suspicious UAVs either by jamming or not, thus the power consumption increases as time goes on. Power consumptions are not compared with each other under different distances, because in each simulation, UAVs fly at random speeds (e.g., random ∆v), thus causing different power consumptions that cannot simply be compared with each other.

**Figure 7:**
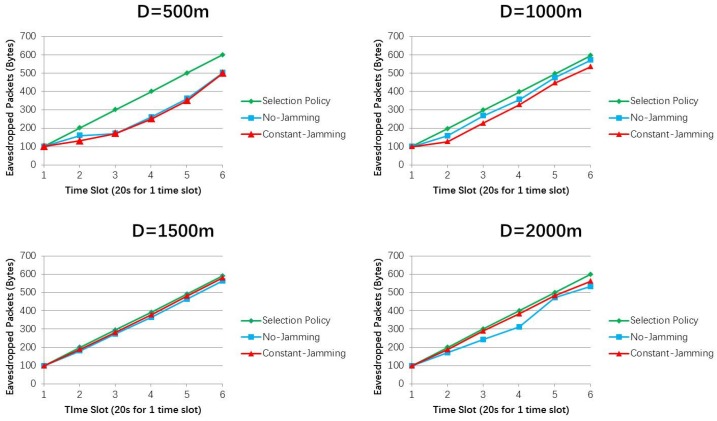
Eavesdropped packets by *UAV_L_* in different Ds with different jamming methods.

Figure 7 presents the other two methods with optimal solutions in terms of the eavesdropped packets. Selection policy outperforms No-Jamming and Constant-Jamming schemes under different distances in the simulations. The reason is that selection policy purposely adapts the jamming power of *UAV_L_* to change the suspicious communication (e.g., to a smaller data rate) for overhearing more packets. In each eavesdropping time slot, *UAV_L_* selects proper eavesdropping case according to the selection policy, thus eavesdropping more information. When D = 500 m, selection policy outperforms the other two schemes by nearly 1.2 times. However, the divisions between the selection policy and the other two methods are narrowed when distances increase. That is because in such long-distance cases, channel conditions dominate the data rate rather than eavesdropping methods, so *UAV_L_* can receive almost the same number of eavesdropped packets regardless which algorithm *UAV_L_* has chosen.

### 5.3. Impact of Typical Fading Models

We apply selection policy into four typical fading channel models, i.e., Rayleigh, Ricean, Weibull and Nakagami, to study the impacts. Each fading channel is characterized with a specific coefficient component. In particular, the coefficient component of Rayleigh, Rician, Weibull, and Nakagami is set to 2, 1, 2, and 0.5, respectively [30].

**Figure 8:**
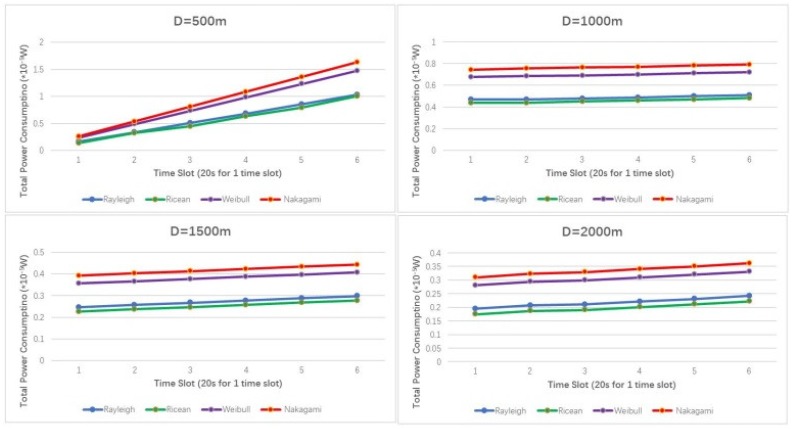
Total power consumptions by *UAV_L_* in different Ds under different fading channels.

**Figure 9:**
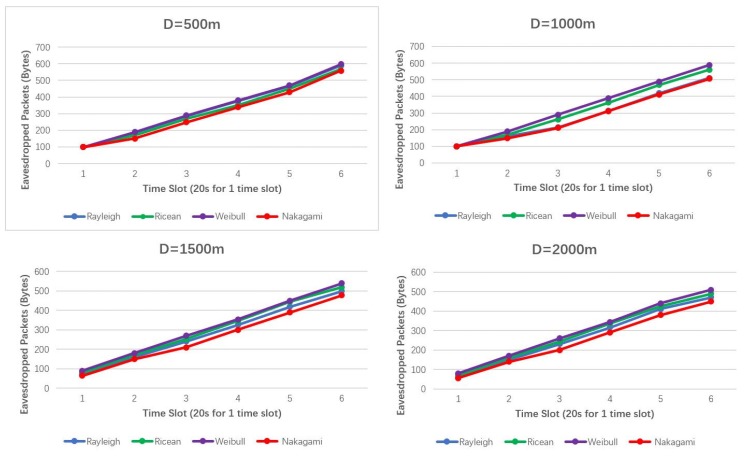
Eavesdropped packets by *UAV_L_* in different Ds under different fading channels.

In Figure 8, total power consumption increases with time going on regardless of distances. However, power consumption increases more sharply in short-distance cases (D = 500 m). That is because in short-distance cases, eavesdropping algorithms dominate eavesdropping performances, while in long-distance cases, fading channels dominate power consumptions rather than eavesdropping algorithms. This can also be interpreted by the eavesdropped packets in regards to the time slots, which is shown in Figure 9.

Figure 9 shows that eavesdropped packets under selection policy linearly grows with time in the four typical fading channels. Selection policy performs best in Weibull fading channel, but not obviously. Total eavesdropped packets are less in Nakagami fading channel than in other three channels with different time slots. This is because Weibull distribution is typically descriptive of channel fading with a dominant line-of-sight (LOS) propagation [41,42], which leads to a small amount of time the channel remains in a fade. For Nakagami channel with the coefficient component of 0.5, the received signal consists of a large number of noise waves with randomly distributed amplitudes, phase, and angles of arrival, which causes distortion and fading of the received signal.

## 6. Conclusions

In this paper, we investigated a proactive eavesdropping and jamming scenario which include four cases for *UAV_L_* to fulfil surveillance tasks. In such a surveillance paradigm, we formulated a power-efficient eavesdropping and jamming problem which has acceptable computing complexity and can be solved. Then, we proposed a selection policy for *UAV_L_* to allocate eavesdropping and jamming power efficiently. Particularly, *UAV_L_* selects the most efficient case for eavesdropping and jamming suspicious UAVs according to the selection policy in each time slot. With such policy, *UAV_L_* can eavesdrop more data by consuming less power. Simulation results showed that selection policy outperformed No-Jamming and Constant-Jamming schemes in both power consumption and data reception. Moreover, we applied selection policy into four typical fading channels to validate the performance, results showed that selection policy performs better in Weibull fading channels in terms of the package received rate (PRR). For future works, we plan to study the problems about jamming and eavesdropping towards suspicious UAV groups, which is a challenge for eavesdropping and jamming policy selection.

## Figures and Tables

**Table 1:** Notations and variables.

Variables	Descriptions
$P_{L}\left(x\right)$	Legitimate monitor consuming power ($P_{E}\left(x\right)+P_{J}\left(x\right)$) at time slot *x*
$P_{E}\left(x\right)$	Legitimate monitor eavesdropping power at time slot *x*
$P_{J}\left(x\right)$	Legitimate monitor jamming power at time slot *x*
$\gamma_{e}\left(x\right)$	SNR of eavesdropping link at time slot *x*
$\gamma_{s}\left(x\right)$	SNR of suspicious link at time slot *x*
$K_{1},K_{2}$	Two constants relating to the channel
$N_{0}$	Power of white Gaussian noise
$d_{1}\left(x\right)$	Distance between *UAV_L_* and *UAV_ST_* at time slot *x*
$d_{2}\left(x\right)$	Distance between *UAV_L_* and *UAV_SR_* at time slot *x*
$P_{L}^{max}$	Maximum consuming power of *UAV_L_*
$P_{L}^{total}$	Total jamming power of *UAV_L_*
n	Gaussian random number
$\alpha_{1},\alpha_{2}$	Path-loss exponent of wireless channel
λ	Coefficient considered to adjust the weights of the autocorrelated component and independent component
δ	SINR/SNR threshold
ρ(x)	Adaptive modulation and coding (AMC) rate at time slot x
ϵ	The required instantaneous bit error rate

**Table 2:** Simulation Parameters.

Parameters	Values
$K_{1}$	0.2
$K_{2}$	3
$\beta_{0}$	2.6
$\beta_{1}$	1
φ	60
𝜑v	[−10, 10]
θ	[0, π]
f	20
l	10
ϵ	0.05
$N_{0}$	3.98 × 10^−12^ W
b	100 bytes
δ	3
λ	0.3
n	0.005377
$\alpha_{1}$	3
$\alpha_{2}$	2.5
D	500 m, 1000 m, 1500 m, 2000 m
$P_{L}^{max}$	8 × 10^−6^ W
ρ	1, 2, 4, 8
Constant Jamming Power	10^−8^ W

## References

[1] Li C., Xu Y., Xia J., Zhao J. (2018). Protecting secure communication under UAV smart attack with imperfect channel estimation. IEEE Access.

[2] Zou Y., Zhu J., Wang X., Hanzo L. (2016). A survey on wireless security: Technical challenges, recent advances and future trends. Proc. IEEE.

[3] Ju H., Zhang R. (2014). Throughput maximization in wireless powered communication networks. IEEE Trans. Wirel. Commun..

[4] Xu J., Zhang R. (2014). Energy beamforming with one-bit feedback. IEEE Trans. Signal Process..

[5] Xu J., Liu L., Zhang R. (2014). Multiuser MISO beamforming for simultaneous wireless information and power transfer. IEEE Trans. Signal Process..

[6] Xu J., Zhang R. (2016). A general design framework for MIMO wireless energy transfer with limited feedback. IEEE Trans. Signal Process..

[7] Tran H., Zepernick H.J. Proactive attack: A strategy for legitimate eavesdropping. Proceedings of the IEEE International Conference on Communications and Electronics (ICCE).

[8] Zeng Y., Zhang R. (2016). Wireless information surveillance via proactive eavesdropping with spoofing relay. IEEE J. Sel. Top. Signal Process..

[9] Ayub M.F., Ghawash F., Shabbir M.A., Kamran M., Butt F.A. Next Generation Security and Surveillance System Using Autonomous Vehicles. Proceedings of the 2018 Ubiquitous Positioning, Indoor Navigation and Location-Based Services (UPINLBS).

[10] Xu J., Duan L., Zhang R. (2017). Surveillance and intervention of infrastructure-free mobile communications: A new wireless security paradigm. IEEE Wirel. Commun..

[11] Xu J., Duan L., Zhang R. (2017). Proactive eavesdropping via cognitive jamming in fading channels. IEEE Trans. Wirel. Commun..

[12] Zhou X., Maham B., Hjorungnes A. (2012). Pilot contamination for active eavesdropping. IEEE Trans. Wirel. Commun..

[13] Wang X., Li K., Kanhere S.S., Li D., Zhang X., Tovar E. PELE: Power efficient legitimate eavesdropping via jamming in UAV communications. Proceedings of the Wireless Communications and Mobile Computing Conference (IWCMC).

[14] Lakshmanan S., Tsao C., Sivakumar R., Sundaresan K. Securing wireless data networks against eavesdropping using smart antennas. Proceedings of the 28th International Conference on Distributed Computing Systems.

[15] Raymond R., Midkiff S. (2008). Denial-ofservice in wireless sensor networks: Attacks and defenses. IEEE Perv. Comput..

[16] Kannhavong B., Nakayama H., Nemoto Y., Kato N., Jamalipour A. (2007). A survey of routing attacks in mobile ad hoc networks. IEEE Wirel. Commun..

[17] Meyer U., Wetzel S. A man-in-themiddle attack on UMTS. Proceedings of the 3rd ACM Workshop Wireless Security.

[18] Ohigashi T., Morii M. A practical message falsification attack on WPA. Proceedings of the Joint Workshop Inf. Security.

[19] Shiu Y.-S., Chang S.Y., Wu H.-C., Huang S.C.-H., Chen H.-H. (2011). Physical layer security in wireless networks: A tutorial. IEEE Wirel. Commun..

[20] Christof P., Pelzl J., Preneel B. (2009). Understanding Cryptography: A Textbook for Students and Practitioners.

[21] Elliott C. (2004). Quantum cryptography. IEEE Secur. Priv..

[22] Cui M., Zhang G., Wu Q., Ng D.W.K. (2018). Robust trajectory and transmit power design for secure UAV communications. IEEE Trans. Veh. Technol..

[23] Zhou Y., Yeoh P.L., Chen H., Li Y., Hardjawana W., Vucetic B. Secrecy outage probability and jamming coverage of UAV-enabled friendly jammer. Proceedings of the 11th IEEE Australia International Conference on Signal Processing and Communication Systems (ICSPCS).

[24] Wang Q., Chen Z., Mei W., Fang J. (2017). Improving physical layer security using UAV-enabled mobile relaying. IEEE Wirel. Commun. Lett..

[25] Zhang S., Zhang H., He Q., Bian K., Song L. (2018). Joint trajectory and power optimization for UAV relay networks. IEEE Commun. Lett..

[26] Mukherjee A., Swindlehurst A.L. Optimal strategies for countering dual-threat jamming/eavesdropping-capable adversaries in mimo channels. Proceedings of the Military Communications Conference.

[27] Li Y., Xiao L., Dai H., Poor H.V. Game theoretic study of protecting MIMO transmissions against smart attacks. Proceedings of the IEEE International Conference on Communications (ICC).

[28] Mukherjee A., Swindlehurst A.L. (2013). Jamming games in the MIMO wiretap channel with an active eavesdropper. IEEE Trans. Signal Process..

[29] Yoo T., Goldsmith A. (2006). Capacity and power allocation for fading MIMO channels with channel estimation error. IEEE Trans. Inf. Theory.

[30] Edman M., Kiayias A., Yener B. On passive inference attacks against physical-layer key extraction?. Proceedings of the Fourth European Workshop on System Security, ACM.

[31] Mitrpant C., Vinck A., Luo Y. (2006). An achievable region for the Gaussian wiretap channel with side information. IEEE Trans. Inf. Theory.

[32] Negi R., Goel S. Secret communication using artificial noise. Proceedings of the IEEE International Conference on Vehicular Technology (VTC).

[33] Bloch M., Barros J., Rodrigues M.R.D., McLaughlin S.W. (2008). Wireless information-theoretic security. IEEE Trans. Inf. Theory.

[34] Zheng G., Krikidis I., Li J., Petropulu A.P., Ottersten B. (2013). Improving physical layer secrecy using fullduplex jamming receivers. IEEE Trans. Signal Process..

[35] Wu Q., Mei W., Zhang R. (2019). Safeguarding Wireless Network with UAVs: A Physical Layer Security Perspective. arXiv.

[36] Li A., Wu Q., Zhang R. (2018). UAV-enabled cooperative jamming for improving secrecy of ground wiretap channel. IEEE Wirel. Commun. Lett..

[37] Zhang G., Wu Q., Cui M., Zhang R. (2019). Securing UAV communications via joint trajectory and power control. IEEE Trans. Wirel. Commun..

[38] Li K., Ni W., Wang X., Liu R.P., Kanhere S.S., Jha S. (2016). Energy-efficient cooperative relaying for unmanned aerial vehicles. IEEE Trans. Mobile Comput..

[39] Schoel W.M., Schürch S., Goerke J. (1994). The captive bubble method for the evaluation of pulmonary surfactant: Surface tension, area, and volume calculations. Biochim. Biophys. Acta (BBA)-Gen. Subj..

[40] Boyd S., Vandenberghe L. (2004). Convex Optimization.

[41] Wu Q., Zhang R. (2018). Common throughput maximization in UAV-enabled OFDMA systems with delay consideration. IEEE Trans. Commun..

[42] Wu Q., Zeng Y., Zhang R. (2018). Joint trajectory and communication design for multi-UAV enabled wireless networks. IEEE Trans. Wirel. Commun..

